# Vigorous physical activity and self-rated health during adolescence: A cross-sectional survey

**DOI:** 10.3389/fpubh.2022.961361

**Published:** 2022-10-21

**Authors:** Yongkang Wang, Weizhong She, Guijun Chi, Junsheng Wang

**Affiliations:** ^1^School of Physical Education and Sport Science, Fujian Normal University, Fuzhou, China; ^2^Hebei Vocational College for Correctional Police, Handan, China; ^3^Department of Physical Education, Tangshan Normal University, Tangshan, China; ^4^Institute of Physical Education and Training, Capital University of Physical Education and Sports, Beijing, China

**Keywords:** vigorous physical activity, self-rated health, adolescence, HBSC, survey

## Abstract

**Background:**

Despite the positive relationship between self-rated health and general physical activity, very little research has touched upon the relationship between self-rated health and vigorous physical activity. Such research will help to promote self-rated health among adolescents by addressing the close relationship between self-rated health and the components of physical activity.

**Purpose:**

In this study, the relationship between self-rated health and vigorous physical activity among Chinese adolescents were analyzed.

**Methods:**

The current study was based on a collaborative survey conducted by the WHO in several nations, and the data generated by Health Behavior in School-aged Children every 4 years on health behavior were adopted. The school class was taken as a basic unit for sampling. Cluster sampling was performed systematically, and the possibility was proportional to the population. The sampling was conducted to collect typical cases. The class teachers were informed of the sampling activities, and they were to collect data on the students by distributing the questionnaires.

**Results:**

The research included 116,828 respondents from 36 countries. Among them, 51.82% were girls and 48.18% were boys. A total of 98.54% of the respondents considered their health status as fair or above when completing the questionnaire, while 1.46% believed that their health status was poor. Moreover, more than 86% of the respondents participated in vigorous exercise more than weekly, and better self-health ratings were found among respondents who exercised more than weekly (once a week: OR = 1.95, CI: 1.86–2.04; two or three times a week: OR = 1.69, CIL: 1.63–1.76; four or six times a week: OR = 1.30, CI: 1.25–1.35). Certain respondents reported better results for self-rated health (4–6 h every week: OR = 1.36, CI: 1.30–1.43; 2 or 3 h every week: OR = 1.48, CI: 1.42–1.55; 1 h every week: OR = 1.64, CI: 1.57–1.72).

**Conclusion:**

In this study, empirical evidence is provided for the relationship between self-rated health and vigorous physical activity among adolescents. From the results, it can be observed that there is a positive relationship between self-rated health and vigorous physical activity among adolescents.

## Introduction

Self-rated health (SRH) has been identified as a standard measure for evaluating perceived health status in public health surveys (1). SRH refers to one's perception of one's overall health status, physical health and mental health (2, 3). SRH is usually measured by using a five- or four-point Likert Scale, which can indicate one's perceived health status (2). According to the World Health Organization (WHO), SRH is more important than traditional morbidity and mortality in evaluating health outcomes in adolescents (4–7). SRH consists of several components, such as general physical functioning, psychological health and health behaviors (8). In numerous previous studies, SRH has been proved to be an important indicator of several health outcomes, including body fatness (9), mental health (10, 11), morbidity (12) and mortality (13, 14). Accordingly, a high prevalence of poor SRH has been well-documented in previous studies (15–17). For example, a population-based survey indicated that 11% of participants rated their health as poor and a decline in SRH with age was found among adolescents residing in urban areas (15). In another large sample survey, 20.9% of Iranian adolescents rated their SRH as poor (17). However, data from a longitudinal cohort study demonstrated that a generally high (%) SRH was reported in Swedish adolescents, especially in boys (18). Furthermore, gender differences in SRH were also found in the previous studies, where boys reported higher SRH than girls (15, 18). By contrast, one Chinese cross-sectional study revealed that the gender differences in SRH were not significant among Chinese adolescents (19). In addition, data from the Health Behavior in School-aged Children (HBSC) survey indicated that more adolescents reported higher SRH and boys rated their health as better than girls.

The positive association between health-related behaviors such as physical activity (PA) and health outcomes has been well-documented in previous studies (20–23). PA is an important predictor of self-perceived health in adolescents (24). Thus, results from a review study showed that most of the included cross-sectional studies reveal that high PA is associated with better SRH in adolescents (8, 25). Furthermore, only four studies did not report a significant association between physical activity and SRH in children and adolescents, which can be explained by the limitations of the studies' designs (8). In addition, a dose-response association between PA and SRH was reported in this review, which indicated that higher PA is linked with higher SRH (8). For example, data from one cross-sectional study indicated that, compared with adolescents recognized to be physically inactive, those adolescents who participated in PA every day were over 11-times more likely to rate their perceived health as “good or excellent” (OR: 11.5; 95% CI: 2.0–65.8) (26). Similarly, one Canadian study also revealed that more physically active adolescents were more likely to report “very good or excellent” SRH than their inactive peers (11). A longitudinal study also reported similar findings (3, 27). For instance, a prospective study (with 4 years of follow-up) revealed that adolescents with insufficient PA tended to rate their perceived health as “poor” (3, 28).

There are an increasing number of researchers showing that an increase in PA from moderate to vigorous plays a critical role in preventing non-communicable diseases (NCDs), such as type II diabetes, cardiovascular disease, obesity and some cancers (29–31). Despite the positive associations between overall PA and SRH, few studies have focused on the relationship between vigorous PA (VPA) and SRH. Identifying strong associations between different types of PA and SRH could be beneficial for the promotion of intense PA in adolescents. Therefore, the present study aimed to explore the association between VPA and SRH in adolescents using public data.

## Methods

### Study participants and design

For the implementation of “Health Behavior in School-aged Children” (HBSC), a cross-sectional study was conducted and relevant research was performed according to the international HBSC protocol (32). This was a collaborative survey by the WHO that was conducted across different nations, and data on health behavior were collected using the HBSC regularly, that is, every 4 years. The samples are representative of adolescents aged between 11 and 15 years in the nation. The class at the school was considered a basic sampling unit. The cluster sampling was systematic, and the probability was proportional to the population. The sampling was performed to obtain samples that were representative of the nation. The teachers of the classes to be sampled were informed, and they collected information on the students by sending them information sheets. There was a brief introduction to the survey on the information sheets, and the students gave the sheets to their guardians or parents. If students refused to take the survey, then they were required to sign opt-forms and give them back. The data were collected anonymously, and standard measures were also taken to protect the students' private information. The teachers responsible for the survey were well-trained, and they sent a self-compiled, anonymous and standard questionnaire to the respondents. The specific information on the protocol, the theoretical framework and the purpose of the international research are described elsewhere (33, 34). Based on the survey performed in 2013 and 2014, the feedback rate was 85% and above in most of the schools and classes and among most of the students (35). The data were collected randomly, and the respondents were representative of the nation; there were 108,666 girls and 105,414 boys.

### Measures

#### Vigorous physical activity (independent variables)

To measure VPA, the two questions below were asked. The first one was “how often did you do a workout by which you sweat or get out of breath after class?” The answers included never, less than once a month, once a month, once a week, 2 to 3 times a week, 4 to 6 times a week and every day. The second one was “how long did you spend on a workout after class by which you sweat or get out of breath?” The answers included about 7 h or more, about 4 to 6 h, about 2 to 3 h, about 1 h, about half an hour and none. The reliability and validity of the questions were tested several times and determined to be good, as described elsewhere (36, 37).

#### Self-rated health

Self-rated health was assessed using a Likert response scale, with a single question — “Would you say your health is…?”—and a four-point response scale was used including the categories excellent, good, fair and poor. SRH is a standardized indicator that has frequently been used in various areas of health research (38, 39).

#### Covariates

Information on the study participants' sex, age and country were included as covariates, in line with previously published studies (40, 41). These variables were adjusted for further statistical analysis.

### Statistical analysis

Statistical Package Social Science (SPSS) version 23.0 was used for all the statistical analyses in this study. Descriptive analysis was used to report the sample characteristics; percentages and means with standard deviations are used to describe categorical and continuous variables. An ordinal regression model within a generalized linear model was used to establish the association between independence and outcome. After excluding missing cases of variables included in this study, 116,828 samples were included for final analysis. Parameters on model fit and diagnosis were also reported. The significance of the statistical test was set up as *p* < 0.05.

## Results

Table 1 presents the sample characteristics of this study. According to Table 1, 116,828 participants from 36 countries were involved in this research, of which 48.18% were boys and 51.82% were girls. In terms of age composition, adolescents aged 15 made up the largest proportion of the whole sample, at 37.10%. This was closely followed by the group of adolescents aged 13, which accounted for 35.08%, while adolescents aged 11 comprised the smallest proportion of the sample, at 27.83%. Table 2 depicts the participants' results regarding their SRH and the frequency and duration of VPA per week.

**Table 1:** Sample characteristics of this study.

	**Proportion**
**Country**	
Albania	3.12%
Armenia	1.25%
Austria	2.12%
Belgium (Flemish)	2.73%
Bulgaria	2.91%
Canada	6.50%
Switzerland	4.44%
Czech Republic	3.48%
Germany	3.40%
Denmark	2.41%
Estonia	2.65%
England	1.49%
Spain	4.22%
France	3.37%
Greece	2.97%
Croatia	3.14%
Hungary	2.65%
Ireland	0.72%
Israel	1.04%
Iceland	6.24%
Italy	2.65%
Luxembourg	1.73%
Latvia	4.02%
Republic of Moldova	3.82%
Makedonski denar	1.21%
Malta	0.88%
Netherlands	2.56%
Norway	0.28%
Poland	3.23%
Portugal	3.60%
Romania	1.67%
Russian Federation	2.94%
Scotland	1.26%
Sweden	4.38%
Slovenia	3.42%
Wales	1.48%
**Sex**	
Boy	48.18%
Girl	51.82%
**Age category**	
11	27.83%
13	35.08%
15	37.10%

**Table 2:** Descriptive results for outcome and independents.

	**Proportion**
**Self-rated health**	
Excellent	36.36%
Good	50.80%
Fair	11.39%
Poor	1.46%
**Vigorous physical activity frequency**	
Every day	17.94%
4–6 times a week	25.12%
2–3 times a week	30.08%
Once a week	13.39%
Once a month	3.83%
Less than once a month	4.69%
Never	4.94%
**Vigorous physical activity hours a week**	
7 h and more	11.38%
4–6 h	15.27%
2–3 h	26.40%
1 h	22.80%
Half an hour	14.76%
None	9.38%

As shown in Table 2, 98.54% of the adolescents in the study gave a fair or above rating for their health, while only 1.46% of the adolescents rated their health as poor. In addition, more than 86% of the adolescents engaged in vigorous exercise at least once a week. Meanwhile, 13.46% of the adolescents had conducted vigorous exercise no more than once a month, while 4.94% had never carried out VPA. Furthermore, over 90% of the adolescents did at least half an hour of VPA per week, with the majority choosing to exercise for 2–3 h a week.

Table 3 reveals the relationship between the frequency and duration of VPA and SRH among adolescents. Generally, worse SRH was observed among participants who participated in less VPA in terms of both frequency and hours (once a week: OR = 1.95, CI: 1.86–2.04; 2–3 times a week: OR = 1.69, CI: 1.63–1.76; 4–6 times a week: OR = 1.30, CI: 1.25–1.35). In addition, less vigorously active adolescents were more likely to report worse SRH (4–6 h per week: OR = 1.36, CI: 1.30–1.43; 2–3 h per week: OR = 1.48, CI: 1.42–1.55; 1 h per week: OR = 1.64, CI: 1.57–1.72) compared with those reporting 7 h or more. The results for the covariates included in the model were also shown in Table 3.

**Table 3:** Results for the associations between vigorous physical activity and self-rated health.

	**OR**	**95% CI**		***p*-value**
**Frequency (ref** **=** **Every day)**				
4–6 times a week	1.30	1.25	1.35	0.000
2–3 times a week	1.69	1.63	1.76	0.000
Once a week	1.95	1.86	2.04	0.000
Once a month	2.14	2.00	2.29	0.000
Less than once a month	2.29	2.15	2.45	0.000
Never	2.08	1.94	2.24	0.000
**Hours (ref** **=** **7 h or more)**				
4–6 h	1.36	1.30	1.43	0.000
2–3 h	1.48	1.42	1.55	0.000
1 h	1.64	1.57	1.72	0.000
Half an hour	1.72	1.63	1.81	0.000
None	1.62	1.52	1.73	0.000
**Sex (ref** **=** **girls)**				
Boys	1.43	1.40	1.46	0.000
**Age (ref** **=** **11 years)**				
15 years	1.74	1.69	1.79	0.000
13 years	1.41	1.37	1.45	0.000
Country	1.01	1.009	1.01	0.000

OR, odd ratio; CI, confidence interval; ref, reference group.

## Discussion

The main aim of the present study was to analyse the association between VPA and SRH in adolescents, using public data based on a large sample size from the HBSC survey in 42 European countries. This study mainly that after controlling for some significant covariates, there was a significant association between VPA and SRH in adolescents. More specifically, more participations in VPA and more durations of VPA were both two potential contributors to adolescents' SRH. More analysis is displayed below.

The current study indicated that adolescents who reported higher levels of VPA were more likely to rate better perceptions of health. This research finding was consistent with the previous studies. Another study (42) investigated the relationship between VPA and SRH. Similarly, another study found a positive correlation between more participation in PA at higher intensity, further demonstrating the roles of VPA on SRH outcomes in adolescents. Collectively, the current study, alongside previous studies, provides new insights into the current limited knowledge of VPA and health promotion research.

Some underlying mechanisms can be used in this study for a better understanding of the roles of VPA on SRH in adolescents. A recent meta-analytical study found that VPA would contribute to physical health outcomes (43), including lower risks of adiposity and cardiovascular diseases. This review suggests that adolescents participating in more VPA are likely to be healthier than those participating in less VPA, making those with higher VPA report better SRH. In addition to physical health outcomes, emerging evidence has suggested that participating in more VPA can to some extent promote mental health and well-being (44, 45). Similar to explaining the roles of VPA on physical health indicators, owing to contributions of VPA to mental health, it is also available that adolescents who reported VPA to display better mental health, then rating better perceived overall health. Although speculative, it is reasonable to assume the positive roles of VPA in the promotion of healthy adolescents, the actual underlying mechanism explaining the association between VPA and SRH should be further clarified.

This study also suggested that adolescents reported participating less in VPA. In comparison to those in a national study, 43.06% of the adolescents reported that they participated in VPA four or more times a week, and this prevalence is similar to that of their European counterparts (ranging from 37% to 57%) (46). Compared with that in another cross-sectional study, however, the prevalence of frequent (four or more times a week) VPA in the present study was lower than that in Icelandic adolescents (64.3%) (47). In addition, 86.54% of the adolescents in the present study participated in VPA more than once a week, which is in line with a previous study (48). In the aforementioned study, 84% of American adolescents engaged in VPA more than once a week (48). Owing to the health benefits of VPA, it should be considered to increase VPA in adolescents.

Compared with previous studies, the current study focused on VPA rather than moderate to vigorous physical activity, which can add new evidence to the literature. To our knowledge, previous studies primarily laid sufficient research interest and attention on MVPA and SRH, while very few studies collected information on VPA in insolation. Our study, based on a large sample size, provides new insights into, advancing the knowledge on the associations between PA at different intensities and health outcomes in adolescents. Considering our preliminary evidence in our study, regarding VPA and SRH, it helps strengthen the current PA guidelines to include more recommendations on PA at a higher intensity. However, the research findings in the present study were based on self-reported measures, which are subject to measurement errors. Thus, research findings concerning the association between VPA and SRH may be potentially influenced. Future studies are promising to use more advanced measures to estimate the associations between VPA and SRH in adolescents.

### Implications

Although the results of the present study cannot provide causal evidence, the positive relationship implies that VPA might be an important contributor to SRH in adolescents. Given the findings from the present study, there is a potential to promote SRH by increasing VPA in adolescents. Future interventional studies should explore how to increase VPA levels in adolescents.

### Study limitation

Several limitations of the present study should be noted. First, the data included in the analysis were from a cross-sectional study, which might not provide a causal explanation for the association between VPA and self-rated health in adolescents. The second limitation is the use of self-administrated measurements, which may have contributed to the overestimation of VPA and self-rated health in adolescents.

## Conclusion

This study provides empirical evidence for the association between VPA and self-rated health in adolescents and indicates that VPA is positively associated with self-rated health in adolescents. For efficient health promotion in adolescents, it would be better to encourage adolescents to participate in more VPA.

## Data availability statement

The original contributions presented in the study are included in the article/supplementary material, further inquiries can be directed to the corresponding author.

## Ethics statement

All data were anonymized and publicly available; therefore no ethical approval was required. Written informed consent to participate in this study was provided by the participants' legal guardian/next of kin.

## Author contributions

YW and JW: writing—original draft. JW and GC: formal analysis. YW, JW, GC, and WS: writing—review and editing. All authors contributed to the article and approved the submitted version.

## Funding

This study was supported by Beijing Social Science Foundation for Youths (project number: 19YTC033): Study on Correlation between Motor Ability and Physical Health Status of Primary School Students.

## Conflict of interest

The authors declare that the research was conducted in the absence of any commercial or financial relationships that could be construed as a potential conflict of interest.

## Publisher's note

All claims expressed in this article are solely those of the authors and do not necessarily represent those of their affiliated organizations, or those of the publisher, the editors and the reviewers. Any product that may be evaluated in this article, or claim that may be made by its manufacturer, is not guaranteed or endorsed by the publisher.
